# Supplementary material for: Individualised electrophysiological neural field models for the assessment of thalamocortical function in disorders of consciousness: A multicentre study
Source: PLoS Comput Biol. 2026 Oct 28;22(10):e1014905. doi: 10.1371/journal.pcbi.1014905 (PMC13730432; doi:10.1371/journal.pcbi.1014905)
Supplement: Supplementary file 1 [file pcbi.1014905.s003.pdf]

We would like to thank both reviewers for their constructive comments on the manuscript, which we believe have helped to greatly improve the overall quality of the paper. We have revised the manuscript accordingly, and we hope we have addressed all of your concerns satisfactorily.

Reviewer 1

Comment 1

The current version of Figure 1 is very small... Maybe putting panels A and B one below the other could help readability? I also feel that panel B could be more explicit on model details thus allowing the reader to have a better idea of what kind of model we are talking about without the need to go and look in the Methods.

We agree with the reviewer that in its current size Figure 1 is small and cramped, especially for a standard PDF article format. To address this, we have placed panels A and B in two rows to improve overall readability. We have also expanded panel B, adding further explanations of the parameters shown on the figure, in order to make the model structure easier to understand and help the summary figure stand on its own. We include the new figure here for the reviewer's convenience:

Figure 1. A Protocol of the study. High-density EEG recordings from patients with prolonged DoC were used to estimate the parameters of a corticothalamic neural field model. To this end, the average power spectrum of 183 scalp channels, as well as the average from electrodes Fz, Pz and surrounding electrodes, were obtained. After fitting the model, time series were simulated for all subjects using the parameters obtained from the three power spectra. The Lempel-Ziv complexity of the empirical and simulated time series was obtained, their correlation was computed and their capacity to replicate group differences was assessed. **B Corticothalamic neural field model.** The cortex component consists of an excitatory population (e) and an inhibitory population (i); the thalamus component consists of a reticular population (r) and a relay population (s). The relay population receives external inputs denoted by n. The non-zero gains between each two populations are denoted by G_{ab}. Inhibitory connections are shown in dashed lines. There is no time delay between populations within the same anatomical part, but a loop delay of t_0 exists for any connection between cortex and thalamus.

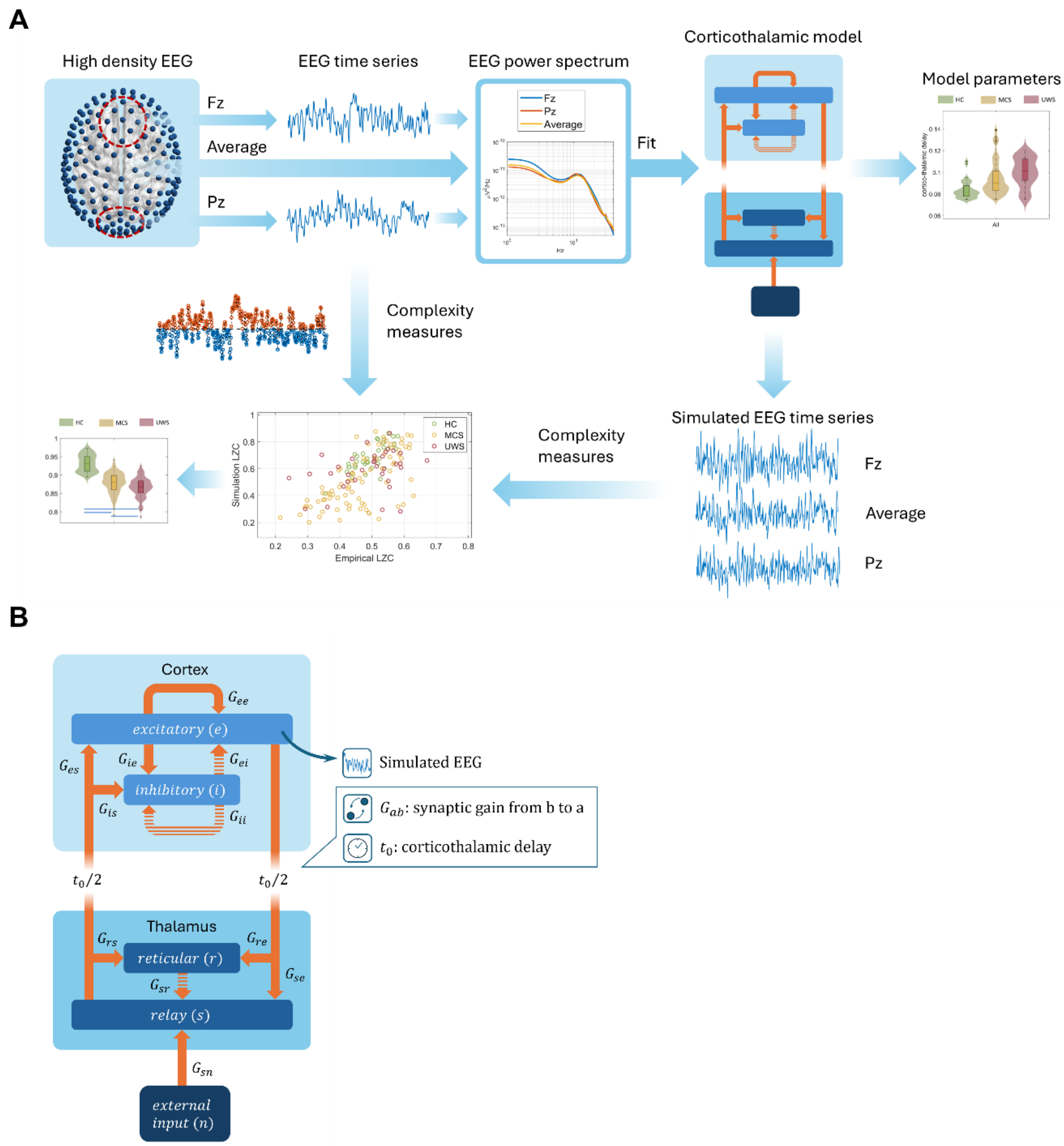

Comment 2

In the Discussion the authors say that "the corticothalamic model [...] captured key spectral features" but from Figure 2 and the corresponding text in the results I do not see adequate numerical justification to this. I would like to see a more detailed treatment of the salient reproduced features mentioned in discussion. This holds also for other observations throughout the discussion, like comments on high fit parameter standard deviations etc.

Following on this point, it is not obvious to me how to interpret the spectra in Figure 2. More detailed explanation of the difference between "spectral model" and "timeseries" and the important different between "timeseries" and the other shown spectra should be added in the

main text, in particular in the Results section. Again, more granular numerical results than the relatively "abstract" Chi square values would be very helpful.

We think the reviewer makes a good point about the lack of proper numerical justification for the claim that the model captured key spectral features. To address this, new analyses have been added in the Results section, quantifying the reproduction of spectral features. Specifically, we have compared the empirical EEG data's spectra, the fitted spectral model, and the spectra computed from simulated time series in terms of alpha peak frequency and relative power across frequency bands. These analyses are now reported in the Results section and summarized in Table S1, as follows:

> "Table S1 listed the spectral features of the empirical and fitted power spectra. Alpha and beta peak frequencies were quantified only in the healthy control group because distinct spectral peaks in these ranges were not consistently identifiable in the patient groups. Peak-frequency analyses were therefore restricted to cases where a meaningful local spectral maximum could be defined, whereas relative band-power analyses were performed for all groups. Across theta, delta, alpha, beta and gamma bands, the relative power was well replicated with minimal deviation from the empirical data, as supported by evidence for the null hypothesis in the Bayesian analysis and showing high correlation at individual level, as shown in Table S1. Additionally, Pearson correlation analysis confirmed that the simulated spectra accurately predicted empirical subject-level variations across most features."

We include Table S1 here for your convenience:

Table S1. Comparison of spectral features between empirical, fitted, and simulated timeseries power spectra. Alpha peak frequency was defined as the frequency of the dominant local maximum within the 8–13 Hz range, and beta peak frequency as the frequency of the dominant local maximum within the 13–30 Hz range. These peak frequencies are reported for the HC group. Relative band power (delta, theta, alpha, beta, and gamma) is calculated separately for the HC, MCS, and UWS groups. To assess how accurately the model captures individual subject-level variance, Pearson correlation coefficients between the fitted spectra and real spectra (***r (FS)***), as well as the correlation between the simulated timeseries spectra and real spectra (***r (TS)***) are reported. Highlighted features denote statistically significant differences between the empirical and simulated data, evaluated via Bayesian paired t-tests for both fitted spectra (BF_{10} (FS)) and timeseries spectra (BF_{10} (TS)), showing metrics of where model accuracy is lower. HC: healthy controls; MCS: minimally conscious state; UWS: unresponsive wakefulness syndrome.

		Real Spectra	Fitted Spectra (FS)	Timeseries Spectra (TS)	R (FS)	BF_{10} (FS)	R (TS)	BF_{10} (TS)
		**Liège**						
**HC**	**Alpha peak frequency**	9.581 ± 1.052	9.441 ± 0.876	9.892 ± 0.558	0.91	0.59	0.62	**1.1**
	**Beta peak frequency**	20.004 ± 4.048	17.844 ± 2.034	16.354 ± 3.036	0.56	0.49	0.83	**16**
	**Delta band relative power**	33.610 ± 14.545	33.778 ± 14.88	15.435 ± 8.359	0.99	0.04	0.76	**>30**
	**Theta band relative power**	18.221 ± 5.931	17.285 ± 5.365	14.311 ± 5.323	0.96	0.51	0.78	**>30**
	**Alpha band relative power**	29.718 ± 15.877	30.897 ± 16.410	28.369 ± 13.001	0.99	0.44	0.87	0.29
	**Beta band relative power**	15.341 ± 6.094	14.754 ± 5.893	36.267 ± 13.147	0.97	0.39	0.83	**>30**
	**Gamma band relative power**	3.130 ± 1.819	3.317 ± 1.923	5.655 ± 4.183	0.98	0.44	0.59	**>30**
**MCS**	**Delta band relative power**	59.255 ± 15.308	59.424 ± 15.386	41.884 ± 21.094	0.99	0.25	0.77	**>30**
	**Theta band relative power**	16.211 ± 7.276	16.058 ± 7.227	18.638 ± 7.948	0.99	0.37	0.76	**>30**
	**Alpha band relative power**	7.114 ± 3.976	7.489 ± 3.914	12.636 ± 5.595	0.98	<0.03	0.69	**>30**
	**Beta band relative power**	12.196 ± 9.080	11.559 ± 9.073	21.856 ± 18.991	0.99	<0.03	0.75	**>30**
	**Gamma band relative power**	5.262 ± 4.216	5.523 ± 4.246	5.016 ± 8.645	0.98	**3.50**	0.40	0.12
**UWS**	**Delta band relative power**	58.572 ± 14.760	56.048 ± 9.639	37.671 ± 13.117	0.80	0.58	0.44	**>30**
	**Theta band relative power**	14.223 ± 6.644	17.910 ± 5.941	19.693 ± 4.686	0.69	<0.03	0.56	**>30**
	**Alpha band relative power**	6.792 ± 4.693	8.748 ± 3.600	15.916 ± 3.799	0.81	<0.03	0.47	**>30**
	**Beta band relative power**	13.630 ± 8.59	11.559 ± 5.132	22.847 ± 11.004	0.71	0.98	0.30	**>30**
	**Gamma band relative power**	6.834 ± 4.993	5.793 ± 3.204	3.901 ± 2.598	0.72	0.69	0.24	**>30**
		**Paris**						
**HC**	**Alpha peak frequency**	10.312 ± 0.981	9.706 ± 0.836	10.143 ± 0.567	0.75	**55**	0.57	0.34
	**Beta peak frequency**	19.717 ± 3.706	18.218 ± 3.874	14.854 ± 1.407	0.15	0.41	0.82	**>30**

	**Delta band relative power**	28.690 ± 9.600	28.948 ± 9.849	12.750 ± 5.849	0.99	0.29	0.78	**>30**
	**Theta band relative power**	17.023 ± 4.481	16.498 ± 4.235	13.527 ± 4.308	0.96	**2.30**	0.61	**>30**
	**Alpha band relative power**	32.517 ± 15.332	33.091 ± 15.628	35.336 ± 17.702	0.99	0.66	0.89	**1.4**
	**Beta band relative power**	17.280 ± 5.768	16.703 ± 5.632	34.033 ± 12.389	0.98	**6.50**	0.85	**>30**
	**Gamma band relative power**	4.524 ± 2.723	4.806 ± 2.844	4.384 ± 3.119	0.99	**49**	0.47	0.18
**MCS**	**Delta band relative power**	55.913 ± 14.906	56.555 ± 15.385	41.200 ± 16.186	0.99	<0.03	0.80	**>30**
	**Theta band relative power**	21.437 ± 7.120	20.809 ± 7.836	23.763 ± 8.111	0.96	<0.03	0.41	**>30**
	**Alpha band relative power**	7.831 ± 3.571	8.385 ± 4.003	14.507 ± 4.741	0.99	<0.03	0.86	**>30**
	**Beta band relative power**	10.047 ± 8.017	9.654 ± 7.741	17.484 ± 14.022	0.98	<0.03	0.58	**>30**
	**Gamma band relative power**	4.297 ± 4.580	4.642 ± 4.991	3.066 ± 4.397	0.99	<0.03	0.24	**9.1**
**UWS**	**Delta band relative power**	58.521 ± 15.768	59.150 ± 14.253	44.994 ± 15.889	0.99	0.04	0.72	**>30**
	**Theta band relative power**	18.758 ± 7.621	18.108 ± 6.559	22.292 ± 6.438	0.97	<0.03	0.56	**>30**
	**Alpha band relative power**	7.159 ± 3.659	7.498 ± 3.386	13.402 ± 4.489	0.98	<0.03	0.85	**>30**
	**Beta band relative power**	10.006 ± 8.257	10.100 ± 8.319	16.543 ± 14.155	0.98	<0.03	0.46	**>30**
	**Gamma band relative power**	4.820 ± 4.617	5.195 ± 5.324	2.789 ± 3.423	0.98	<0.03	0.35	**>30**

The added analyses show that the directly fitted spectral model reproduces most empirical spectral features to a satisfactory degree, while the spectra derived from simulated time series show weaker correspondence with the empirical data. Corresponding sections have been added in the methods as well:

Spectral features extraction

“We extracted spectral features from the empirical EEG power spectra, the fitted analytical spectra generated by the model, and the spectra computed from simulated time series using the fitted parameters. For each subject and condition, we extracted relative power in the delta (1-4 Hz), theta (4-8 Hz), alpha (8-13 Hz), beta (13-30 Hz), and gamma bands (30-45 Hz), as well as alpha and beta peak frequency (for the HC only). Relative band

power was calculated as the proportion of total power within the analyzed frequency range accounted for by each frequency band.

[…]

Statistical analyses

The values of G_{ee}, G_{ei}, G_{ese}, G_{esre}, G_{srs}, α, β, t_0, P_{EMG} and empirical and simulated LZC, PE and SE were compared among groups and electrodes by means of non-parametric statistical tests. Kruskal-Wallis tests were used to detect global interactions between the three groups (HC, MCS and UWS), and if interactions were found, Mann-Whitney *U*-tests were performed to determine between-group differences. To account for multiple comparisons, a false discovery rate (FDR) correction was applied [88]. The significance level was set at α=0.05. The correlation strength was interpreted following conventional thresholds: high ($|r| > 0.6$), moderate ($0.6 \geq |r| > 0.4$) and low ($|r| \leq 0.4$). Bayes factors were used to quantify evidence for or against differences in spectral features between empirical and model-derived spectra. $BF_{10} > 1$ indicates evidence favoring the alternative hypothesis, whereas $BF_{10} < 1$ indicates evidence favoring the null hypothesis. Statistical analyses and signal processing were performed using MATLAB (version R2024b Mathworks, Natick, MA).

[…]

The results demonstrated that the corticothalamic model accurately captured key spectral features across groups. It was able to reproduce the frequency of the alpha and beta peak in the HC subjects, the prominent theta band activity in the MCS patients, and the delta-dominated power spectrum in UWS patients

[…]

Having established that the model's parameter space can distinguish clinical groups, we further analyzed the signal complexity of the real and simulated data, providing the link between the abstract parameters and measurable neurophysiological output. The simulated time series did not reproduce all spectral features with the same precision as the fitted spectra, which indicates that the conversion from spectral-domain fitted parameters to time-domain simulation parameters may introduce some loss of precision. Importantly, however, the simulated signals retained the main dynamical features needed for the present analysis. This provides an independent validation of the modelling approach: the model was not fitted to LZC, PE or SE, but the positive results suggest that the fitted parameters capture underlying signal dynamics beyond the power spectrum alone."

Comment 3

Throughout the text, there are a lot of abbreviations that are either defined multiple times, or never used again. I believe that a more coherent use of abbreviations (and maybe the definition of less abbreviations altogether) would benefit readability. Following on this, the results section is at times very dense and a little difficult to follow.

We thank the reviewer for pointing out the problems with the abbreviations throughout the paper. We have thoroughly revised the manuscript and improved the abbreviations to increase the readability: repeated definitions were removed, abbreviations that were not used more than once were deleted.

Comment 4

The introduction and discussion sections are well structured but I am missing a more clear positioning of the presented research. The clinical considerations opening the manuscript suggest me that the final goal of this research may be of translational / clinical interest but I do not see an at least tentative way forward. Of course there is no strong need of being a clinically oriented work to proceed to publication, but I would like to read a clearer declaration of intent: translational / clinical vs. neuroscientific / fundamental.

We thank the reviewer for this important observation. We agree with the reviewer that ints current form, the paper lacked a description of more specific direction for the goals of future related research. We have revised the Introduction, the Discussion and the Conclusion to clarify that the present work is primarily positioned as a mechanistic neuroscience study, motivated by a clinical problem but not intended to provide an immediately applicable clinical diagnostic or prognostic tool. Meanwhile, in the last paragraph of the discussion, we have outlined possible future translational applications of the model, including the simulation of brain stimulation and pharmacological interventions. These discussions together clarify both the current mechanistic focus of the study and its potential value for future clinically oriented research.

> **Introduction**
>
> "In the present study, our primary goal is not to propose an immediately applicable clinical biomarker or therapeutic tool. Rather, we aim to assess whether this modelling framework can provide a biologically interpretable account of altered corticothalamic dynamics in DoC. We aim to leverage two large independent high-density EEG datasets of healthy controls and patients with DoC..."
>
> **Discussion**
>
> "The thalamus is a subcortical structure that serves as a central communication hub between the subcortical regions, the cerebellum, and the cortex… Notably, the central thalamus is critically involved in many cases of DoC, as shown by the fact that direct injury

to this structure can by itself produce disturbances in consciousness... According to the mesocircuit hypothesis, amantadine may be effective by promoting dopaminergic input to the striatum, and thus enhancing thalamocortical synaptic activity. ~~Thus, understanding patient-specific impairments in thalamocortical circuits could be key to enabling targeted interventions on DoC patients aiming at restoring arousal and awareness.~~ Characterizing patient-specific alterations in thalamocortical circuits is important for understanding the neurophysiological mechanisms underlying impaired awareness in DoC. In the long run, such mechanistic knowledge may also inform translational efforts aimed at identifying biomarkers or guiding interventions that target corticothalamic dynamics.

[…]

The previously reported high diagnostic accuracy in discriminating UWS and eMCS patients provided by PE_{θ} in empirical recordings [20,22] is a key motivating factor in the potential use of the model in biomarker development. The present results should be interpreted primarily as mechanistic: they suggest that the model may help explain why condition-specific complexity differences emerge in EEG, rather than establishing the model itself as a clinical biomarker."

Conclusion

"We demonstrated the feasibility of using neural field models to fit high-density EEG recordings from patients with DoC, and their ability to shed light on the specific thalamocortical circuits that contribute to aberrant power spectra. Moreover, we showed the capacity of the model to generate synthetic signals that closely mimic complexity features of the empirical recordings. The model's ability to detect impaired communication between neuronal populations, combined with its capacity to simulate EEG time series, provides a promising mechanistic framework for studying individual impairments in DoC. In future work, the framework may also provide insight into estimation of proximity to criticality, and testing personalised treatment strategies aimed at restoring consciousness."

Comment 5

I would appreciate to see a well defined paragraph on the novelty of the study. The discussion is quite well-written and documented but at times it risks to feel like we already knew / expected most of the nice results in the manuscript.

We thank the reviewer for this suggestion. In the discussion, we now explain that the main novelty is that we use a physiologically informed neural field model to study how corticothalamic network alterations associated with disorders of consciousness can be inferred from scalp EEG spectral features. In addition, we go beyond spectral fitting by testing whether

the model can simulate EEG signals that reproduce complexity features of the empirical recordings. This provides a link between fitted corticothalamic parameters and measurable dynamical properties of EEG signals.

> "The results are also in agreement with those observed using the same model on the EEG of patients experiencing acute coma within the first 48 hours after cardiac arrest, in which patients with poor outcome showed increased corticothalamic inhibition, and higher delay compared to those with good outcome. ~~Our study adds to the literature by further supporting this theory from the perspective of biophysically informed EEG modelling.~~ This represents an important extension of previous EEG-based analyses of DoC. Although the corticothalamic parameters in the model should not be understood as direct measurements of thalamic activity, their group-level variation offers a physiologically grounded account of how altered corticothalamic interactions may contribute to impaired consciousness. In this sense, our study supports the use of biophysically informed EEG modelling as a mechanistic approach for probing circuit-level dysfunction in DoC.
>
> […]
>
> While this result is in agreement with Sitt et al. [20], where it was demonstrated that lower-frequency band complexity is a more robust and sensitive marker for distinguishing levels of consciousness in DoC patients, our study extends this by showing that a biophysical model fitted to EEG spectra can for the most part reproduce condition-specific complexity profiles."

Comment 6

Please pay attention at some typos here and there throughout the manuscript. I have also seen a broken internal link that should be fixed.

We thank the reviewer for bringing this to our attention. We carefully corrected all the typos we could find and fixed the broken link.

Reviewer 2

Comment 1: Modelling

If I understood correctly, the neural field model is fitted to EEG signals averaged across 183 electrodes... The rationale for reducing the dimensionality of the problem is understandable, but the limitations of this simplification should be discussed more explicitly... I think it is important to comment on whether future extensions of the model could incorporate structural connectivity or multi-node thalamo-cortical architectures while still allowing parameter estimation from EEG data.

We agree with the reviewer about the limitations of reducing the whole scalp EEG to a single average value. In the revised manuscript, we have expanded the Limitations and Future Directions section to discuss more explicitly the potential issues of using a dimension-reduced, single-node neural field model. Importantly, we have revised the interpretation of the fitted corticothalamic parameters to avoid overstating their physiological specificity. Following the reviewer's suggestion, we added a paragraph discussing future extensions of the model toward multi-node thalamocortical architectures: a possible first step in which regionally specific EEG signals could be simulated using a model with a shared thalamic population and separate cortical nodes.

> "The results are also in agreement with those observed using the same model on the EEG of patients experiencing acute coma within the first 48 hours after cardiac arrest, in which patients with poor outcome showed increased corticothalamic inhibition, and higher delay compared to those with good outcome. ~~Our study adds to the literature by further supporting this theory from the perspective of biophysically informed EEG modelling.~~ This represents an important extension of previous EEG-based analyses of DoC. Although the corticothalamic parameters in the model should not be understood as direct measurements of thalamic activity, their group-level variation offers a physiologically grounded account of how altered corticothalamic interactions may contribute to impaired consciousness. In this sense, our study supports the use of biophysically informed EEG modelling as a mechanistic approach for probing circuit-level dysfunction in DoC.
>
> […]
>
> Beyond the constraints of the empirical data, the spatial resolution is also limited by our current use of a spatially averaged single-node neural field model. However, future extensions of the model can address this issue directly. Structural connectivity based on diffusion-weighted imaging (Deco 2008, Breakspear 2017) and multi-node thalamocortical architectures could be incorporated into the framework while maintaining parameter estimability from clinical EEG data. A first step in this direction could be simulating regionally specific EEG signals (e.g., at Fz and Pz) using a multi-node model where the excitatory populations of each node are connected to each other while one thalamic population is shared among nodes. Such an approach would allow cortico-cortical and cortico-thalamo-cortical interactions to be studied while preserving the physiological interpretability of the model."

Comment 2: Dataset

Two datasets acquired under substantially different experimental conditions... Since the local-global paradigm is itself designed to probe conscious processing, it is difficult to disentangle

effects related to the experimental condition from effects related to the level of consciousness... this deserves substantially more emphasis earlier in the manuscript, particularly when interpreting between-dataset differences and discussing the generalizability of the results.

We agree with the reviewer that the differences between the two datasets (Local-global paradigm vs. resting state) are important for interpreting the results. In the revised Discussion, regarding the model parameters, we now emphasize earlier that the findings distinguishing MCS from UWS, particularly those more evident in the Paris dataset, may reflect paradigm-dependent neural dynamics rather than only stable differences in consciousness level.

> "Interestingly, we found that corticothalamic delay was shorter in UWS than in MCS. One possible interpretation is that shorter delays in UWS may reflect a breakdown of refined thalamocortical processing, leading to fast, asynaptic, and hyperexcitable physiological activity that is incapable of supporting consciousness. This aligns with evidence showing UWS patients have lower PCI associated with stereotypical responses to TMS-EEG [37], altered fMRI signal propagation [60], and increase in thalamic hemodynamic response found in UWS patients [61]. In contrast, the longer delay in MCS could indicate a partially preserved though potentially slowed thalamocortical circuit, which would allow for some conscious processing in the MCS patients, but the structural damage, such as axonal injury or demyelination [17,62], could lead to slowed signal transmission along thalamocortical fibers, thereby increasing effective delays. When interpreting these findings, the difference in the experimental conditions in two datasets should be factored in. The Paris dataset was acquired during the LG paradigm, which is explicitly designed to probe conscious auditory processing. The fact that the model effectively captures network differences under an active probing paradigm suggests that engaging the network may help unmask the different brain dynamics and conscious states between MCS and UWS patients. This task-dependent performance of the model should also be treated with caution when interpreting the complexity measures discussed in the following section."

Comment 3: Figure

Line 223: I guess that it is Figure 1A that shows a schematic description of the main steps of the study, while Figure 1B shows the model.

Response:

We thank the reviewer for noticing this error. We have corrected the reference to Figure 1A and Figure 1B in the revised manuscript.
