# Supplementary material for: Individualised electrophysiological neural field models for the assessment of thalamocortical function in disorders of consciousness: A multicentre study
Source: PLoS Comput Biol. 2026 Oct 28;22(10):e1014905. doi: 10.1371/journal.pcbi.1014905 (PMC13730432; doi:10.1371/journal.pcbi.1014905)
Supplement: Supplementary file 2 [file pcbi.1014905.s001.docx]

**Table A. Comparison of spectral features between empirical, fitted, and simulated timeseries power spectra.** Alpha peak frequency was defined as the frequency of the dominant local maximum within the 8–13 Hz range, and beta peak frequency as the frequency of the dominant local maximum within the 13–30 Hz range. These peak frequencies are reported for the HC group. Relative band power (delta, theta, alpha, beta, and gamma) is calculated separately for the HC, MCS, and UWS groups. Pearson correlation coefficients between fitted spectra and real spectra (***r (FS)***) and the correlation between timeseries spectra and real spectra (***r (TS)***) are reported to assess how accurately the model captures individual subject-level variance. Highlighted features denote evidence for a difference between the empirical and simulated data, evaluated via Bayesian paired t-tests for both fitted spectra ($\boldsymbol{BF}_{\boldsymbol{10}}$ **(FS)**) and timeseries spectra ($\boldsymbol{BF}_{\boldsymbol{10}}$ **(TS)**), indicating regions of lower model accuracy. HC: healthy controls; MCS: minimally conscious state; UWS: unresponsive wakefulness syndrome.

|  | | **Real Spectra** | **Fitted Spectra (FS)** | **Timeseries Spectra (TS)** | ***R* (FS)** | $\boldsymbol{BF}_{\boldsymbol{10}}$ **(FS)** | **R (TS)** | $\boldsymbol{BF}_{\boldsymbol{10}}$ **(TS)** |
| --- | --- | --- | --- | --- | --- | --- | --- | --- |
|  | | **Liège** | | | | | | |
| **HC** | **Alpha peak frequency** | 9.581 ± 1.052 | 9.441 ± 0.876 | 9.892 ± 0.558 | 0.91 | 0.59 | 0.62 | **1.1** |
|  | **Beta peak frequency** | 20.004 ± 4.048 | 17.844 ± 2.034 | 16.354 ± 3.036 | 0.56 | 0.49 | 0.83 | **16** |
|  | **Delta band relative power** | 33.610 ± 14.545 | 33.778 ± 14.88 | 15.435 ± 8.359 | 0.99 | 0.04 | 0.76 | **>30** |
|  | **Theta band relative power** | 18.221 ± 5.931 | 17.285 ± 5.365 | 14.311 ± 5.323 | 0.96 | 0.51 | 0.78 | **>30** |
|  | **Alpha band relative power** | 29.718 ± 15.877 | 30.897 ± 16.410 | 28.369 ± 13.001 | 0.99 | 0.44 | 0.87 | 0.29 |
|  | **Beta band relative power** | 15.341 ± 6.094 | 14.754 ± 5.893 | 36.267 ± 13.147 | 0.97 | 0.39 | 0.83 | **>30** |
|  | **Gamma band relative power** | 3.130 ± 1.819 | 3.317 ± 1.923 | 5.655 ± 4.183 | 0.98 | 0.44 | 0.59 | **>30** |
| **MCS** | **Delta band relative power** | 59.255 ± 15.308 | 59.424 ± 15.386 | 41.884 ± 21.094 | 0.99 | 0.25 | 0.77 | **>30** |
|  | **Theta band relative power** | 16.211 ± 7.276 | 16.058 ± 7.227 | 18.638 ± 7.948 | 0.99 | 0.37 | 0.76 | **>30** |
|  | **Alpha band relative power** | 7.114 ± 3.976 | 7.489 ± 3.914 | 12.636 ± 5.595 | 0.98 | <0.03 | 0.69 | **>30** |
|  | **Beta band relative power** | 12.196 ± 9.080 | 11.559 ± 9.073 | 21.856 ± 18.991 | 0.99 | <0.03 | 0.75 | **>30** |
|  | **Gamma band relative power** | 5.262 ± 4.216 | 5.523 ± 4.246 | 5.016 ± 8.645 | 0.98 | **3.50** | 0.40 | 0.12 |
| **UWS** | **Delta band relative power** | 58.572 ± 14.760 | 56.048 ± 9.639 | 37.671 ± 13.117 | 0.80 | 0.58 | 0.44 | **>30** |
|  | **Theta band relative power** | 14.223 ± 6.644 | 17.910 ± 5.941 | 19.693 ± 4.686 | 0.69 | <0.03 | 0.56 | **>30** |
|  | **Alpha band relative power** | 6.792 ± 4.693 | 8.748 ± 3.600 | 15.916 ± 3.799 | 0.81 | <0.03 | 0.47 | **>30** |
|  | **Beta band relative power** | 13.630 ± 8.59 | 11.559 ± 5.132 | 22.847 ± 11.004 | 0.71 | 0.98 | 0.30 | **>30** |
|  | **Gamma band relative power** | 6.834 ± 4.993 | 5.793 ± 3.204 | 3.901 ± 2.598 | 0.72 | 0.69 | 0.24 | **>30** |
|  |  | **Paris** | | | | | | |
| **HC** | **Alpha peak frequency** | 10.312 ± 0.981 | 9.706 ± 0.836 | 10.143 ± 0.567 | 0.75 | **55** | 0.57 | 0.34 |
|  | **Beta peak frequency** | 19.717 ± 3.706 | 18.218 ± 3.874 | 14.854 ± 1.407 | 0.15 | 0.41 | 0.82 | **>30** |
|  | **Delta band relative power** | 28.690 ± 9.600 | 28.948 ± 9.849 | 12.750 ± 5.849 | 0.99 | 0.29 | 0.78 | **>30** |
|  | **Theta band relative power** | 17.023 ± 4.481 | 16.498 ± 4.235 | 13.527 ± 4.308 | 0.96 | **2.30** | 0.61 | **>30** |
|  | **Alpha band relative power** | 32.517 ± 15.332 | 33.091 ± 15.628 | 35.336 ± 17.702 | 0.99 | 0.66 | 0.89 | **1.4** |
|  | **Beta band relative power** | 17.280 ± 5.768 | 16.703 ± 5.632 | 34.033 ± 12.389 | 0.98 | **6.50** | 0.85 | **>30** |
|  | **Gamma band relative power** | 4.524 ± 2.723 | 4.806 ± 2.844 | 4.384 ± 3.119 | 0.99 | **49** | 0.47 | 0.18 |
| **MCS** | **Delta band relative power** | 55.913 ± 14.906 | 56.555 ± 15.385 | 41.200 ± 16.186 | 0.99 | <0.03 | 0.80 | **>30** |
|  | **Theta band relative power** | 21.437 ± 7.120 | 20.809 ± 7.836 | 23.763 ± 8.111 | 0.96 | <0.03 | 0.41 | **>30** |
|  | **Alpha band relative power** | 7.831 ± 3.571 | 8.385 ± 4.003 | 14.507 ± 4.741 | 0.99 | <0.03 | 0.86 | **>30** |
|  | **Beta band relative power** | 10.047 ± 8.017 | 9.654 ± 7.741 | 17.484 ± 14.022 | 0.98 | <0.03 | 0.58 | **>30** |
|  | **Gamma band relative power** | 4.297 ± 4.580 | 4.642 ± 4.991 | 3.066 ± 4.397 | 0.99 | <0.03 | 0.24 | **9.1** |
| **UWS** | **Delta band relative power** | 58.521 ± 15.768 | 59.150 ± 14.253 | 44.994 ± 15.889 | 0.99 | 0.04 | 0.72 | **>30** |
|  | **Theta band relative power** | 18.758 ± 7.621 | 18.108 ± 6.559 | 22.292 ± 6.438 | 0.97 | <0.03 | 0.56 | **>30** |
|  | **Alpha band relative power** | 7.159 ± 3.659 | 7.498 ± 3.386 | 13.402 ± 4.489 | 0.98 | <0.03 | 0.85 | **>30** |
|  | **Beta band relative power** | 10.006 ± 8.257 | 10.100 ± 8.319 | 16.543 ± 14.155 | 0.98 | <0.03 | 0.46 | **>30** |
|  | **Gamma band relative power** | 4.820 ± 4.617 | 5.195 ± 5.324 | 2.789 ± 3.423 | 0.98 | <0.03 | 0.35 | **>30** |

**Table B. Statistical comparisons of group effects (HC, MCS, UWS) on model parameters across both datasets.** P-values are FDR-corrected for multiple comparisons (Kruskal-Wallis test). In addition to comparing parameter values across groups for the frontal and parietal channel models separately, we also computed the difference in parameter values between these two models for each subject, and tested group differences on these deltas across nine parameters. No significant group differences were found in any parameter, suggesting that model performance on frontal versus parietal channels did not differ significantly between subject groups. HC: healthy control; MCS: minimally conscious state; UWS: unresponsive wakefulness syndrome.

|  |  |  | $\boldsymbol{G}_{\boldsymbol{ee}}$ | $\boldsymbol{G}_{\boldsymbol{ei}}$ | $\boldsymbol{G}_{\boldsymbol{ese}}$ | $\boldsymbol{G}_{\boldsymbol{esre}}$ | $\boldsymbol{G}_{\boldsymbol{srs}}$ | $\boldsymbol{\alpha}$ | $\boldsymbol{\beta}$ | $\boldsymbol{t}_{\boldsymbol{0}}$ | $\boldsymbol{P}_{\boldsymbol{EMG}}$ |
| --- | --- | --- | --- | --- | --- | --- | --- | --- | --- | --- | --- |
| **Liège** | **Frontal** | $\boldsymbol{\chi}^{\boldsymbol{2}}$ | 3.519 | 3.586 | 44.274 | 36.901 | 34.199 | 5.889 | 0.436 | 17.622 | 33.243 |
|  |  | **p-value** | 0.194 | 0.194 | <0.001 | <0.001 | <0.001 | 0.079 | 0.804 | <0.001 | 0.004 |
|  | **Parietal** | $\boldsymbol{\chi}^{\boldsymbol{2}}$ | 11.295 | 0.308 | 38.845 | 34.707 | 38.677 | 2.726 | 1.869 | 24.066 | 26.105 |
|  |  | **p-value** | 0.005 | 0.857 | <0.001 | <0.001 | <0.001 | 0.329 | 0.442 | <0.001 | <0.001 |
|  | **Difference** | $\boldsymbol{\chi}^{\boldsymbol{2}}$ | 1.471 | 1.824 | 1.112 | 0.941 | 1.058 | 2.201 | 1.622 | 0.020 | 0.338 |
|  |  | **p-value** | 0.803 | 0.803 | 0.803 | 0.803 | 0.803 | 0.803 | 0.803 | 0.990 | 0.950 |
| **Paris** | **Frontal** | $\boldsymbol{\chi}^{\boldsymbol{2}}$ | 0.978 | 6.762 | 60.014 | 63.793 | 55.139 | 4.099 | 3.622 | 14.889 | 8.895 |
|  |  | **p-value** | 0.613 | 0.051 | <0.001 | <0.001 | <0.001 | 0.166 | 0.184 | 0.003 | 0.021 |
|  | **Parietal** | $\boldsymbol{\chi}^{\boldsymbol{2}}$ | 1.416 | 0.678 | 64.148 | 60.946 | 54.129 | 6.789 | 0.726 | 17.840 | 6.573 |
|  |  | **p-value** | 0.633 | 0.713 | <0.001 | <0.001 | <0.001 | 0.056 | 0.713 | <0.001 | 0.056 |
|  | **Difference** | $\boldsymbol{\chi}^{\boldsymbol{2}}$ | 0.258 | 0.728 | 1.882 | 0.576 | 5.791 | 0.219 | 0.804 | 1.609 | 0.164 |
|  |  | **p-value** | 0.921 | 0.921 | 0.921 | 0.921 | 0.498 | 0.921 | 0.921 | 0.921 | 0.921 |

**Table C. Statistical comparisons of the pairwise interactions between groups (UWS, MCS, HC) of the model parameters in two dataset (Mann-Whitney U-test). FDR corrected for the number of comparisons.** HC: healthy control; MCS: minimally conscious state; UWS: unresponsive wakefulness syndrome.

|  |  |  |  | $\boldsymbol{G}_{\boldsymbol{ee}}$ | $\boldsymbol{G}_{\boldsymbol{ei}}$ | $\boldsymbol{G}_{\boldsymbol{ese}}$ | $\boldsymbol{G}_{\boldsymbol{esre}}$ | $\boldsymbol{G}_{\boldsymbol{srs}}$ | $\boldsymbol{\alpha}$ | $\boldsymbol{\beta}$ | $\boldsymbol{t}_{\boldsymbol{0}}$ | $\boldsymbol{P}_{\boldsymbol{EMG}}$ |
| --- | --- | --- | --- | --- | --- | --- | --- | --- | --- | --- | --- | --- |
| **Liège** | **Frontal** | **HC-MCS** | **U-value** | 2156 | 1661 | 3361 | 1026 | 1284 | 2259 | 2428 | 1728 | 1472 |
|  |  |  | **p-value** | 0.532 | 0.001 | <0.001 | <0.001 | <0.001 | 0.834 | 0.677 | 0.003 | <0.001 |
|  |  | **HC-UWS** | **U-value** | 1243 | 1171 | 1790 | 832 | 817 | 1184 | 1279 | 991 | 868 |
|  |  |  | **p-value** | 0.462 | 0.142 | <0.001 | <0.001 | <0.001 | 0.166 | 0.689 | <0.001 | <0.001 |
|  |  | **MCS-UWS** | **U-value** | 5341 | 5172 | 5257 | 4830 | 4795 | 4758 | 5049 | 4950 | 4881 |
|  |  |  | **p-value** | 0.426 | 0.946 | 0.638 | 0.151 | 0.137 | 0.137 | 0.638 | 0.385 | 0.221 |
|  | **Parietal** | **HC-MCS** | **U-value** | 2117 | 1838 | 3448 | 984 | 1229 | 2218 | 2245 | 1753 | 1532 |
|  |  |  | **p-value** | 0.408 | 0.022 | <0.001 | <0.001 | <0.001 | 0.718 | 0.742 | 0.006 | <0.001 |
|  |  | **HC-UWS** | **U-value** | 1137 | 1341 | 1749 | 870 | 819 | 1167 | 1359 | 949 | 921 |
|  |  |  | **p-value** | 0.057 | 0.751 | <0.001 | <0.001 | <0.001 | 0.110 | 0.670 | <0.001 | <0.001 |
|  |  | **MCS-UWS** | **U-value** | 5339 | 5087 | 5231 | 4907 | 4783 | 4926 | 5047 | 4824 | 4952 |
|  |  |  | **p-value** | 0.434 | 0.676 | 0.676 | 0.373 | 0.209 | 0.373 | 0.648 | 0.209 | 0.393 |
| **Paris** | **Frontal** | **HC-MCS** | **U-value** | 4291 | 3414 | 6512 | 1585 | 1692 | 4818 | 4788 | 2805 | 3723 |
|  |  |  | **p-value** | 0.605 | 0.071 | <0.001 | <0.001 | <0.001 | 0.071 | 0.071 | <0.001 | 0.315 |
|  |  | **HC-UWS** | **U-value** | 3833 | 2981 | 6226 | 1379 | 1483 | 4105 | 4405 | 2800 | 3008 |
|  |  |  | **p-value** | 0.965 | 0.016 | <0.001 | <0.001 | <0.001 | 0.492 | 0.118 | 0.003 | 0.017 |
|  |  | **MCS-UWS** | **U-value** | 31742 | 31677 | 35297 | 30224 | 31329 | 31400 | 32486 | 34075 | 30445 |
|  |  |  | **p-value** | 0.383 | 0.383 | 0.051 | 0.051 | 0.286 | 0.286 | 0.857 | 0.286 | 0.064 |
|  | **Parietal** | **HC-MCS** | **U-value** | 3982 | 3860 | 6712 | 1466 | 1787 | 4944 | 4398 | 2765 | 4017 |
|  |  |  | **p-value** | 0.801 | 0.627 | <0.001 | <0.001 | <0.001 | 0.331 | 0.620 | <0.001 | 0.801 |
|  |  | **HC-UWS** | **U-value** | 3539 | 3565 | 6345 | 1425 | 1468 | 4060 | 3890 | 3124 | 3193 |
|  |  |  | **p-value** | 0.504 | 0.504 | <0.001 | <0.001 | <0.001 | 0.587 | 0.900 | 0.022 | 0.040 |
|  |  | **MCS-UWS** | **U-value** | 31711 | 32533 | 34546 | 31583 | 31103 | 30770 | 32052 | 35371 | 30517 |
|  |  |  | **p-value** | 0.417 | 0.895 | 0.112 | 0.395 | 0.190 | 0.112 | 0.594 | 0.044 | 0.112 |

**Fig A. Comparison for the parameters fitted to the power spectrum for the three subject groups across channels of different regions in the Liège dataset.** This figure shows the parameters derived from the corticothalamic model for three groups—healthy controls (HC), minimally conscious state (MCS), and unresponsive wakefulness syndrome (UWS)—across frontal, parietal, and whole-brain EEG channels. A consistent pattern is observed across all regions, with significant differences between healthy controls and patients in the following model parameters: 1) $G_{ese}$, the gain of the excitatory corticothalamic loop was lower in DoC patients; 2) $G_{esre}$, the gain of the inhibitory corticothalamic loop is higher in DoC patients; 3) $G_{srs}$, the gain of the intrathalamic loop was higher in DoC patients; 4) $t_{0}$, the delay between cortical and thalamic regions was longer in DOC patients; 5) the EMG component amplitude was larger in DoC patients.

**Fig B. Comparison for the parameters fitted to the power spectrum for the three subject groups across channels of different regions in the Paris dataset.** Consistent with findings from the Liège dataset, significant group differences were observed in several model parameters when fitting for frontal, parietal and whole-brain recordings: 1) $G_{ese}$, the gain of the excitatory corticothalamic loop was lower in DoC patients; 2) $G_{esre}$, the gain of the inhibitory corticothalamic loop is higher in DoC patients; 3) $G_{srs}$, the gain of the intrathalamic loop was higher in DoC patients; 4) $t_{0}$, the delay between cortical and thalamic regions was longer in DoC patients; 5) the EMG component amplitude was larger in DoC patients. Additionally, $G_{ei}$, the gain between excitatory and inhibitory cortical populations, is higher in patients than in healthy controls for the frontal regions data; the gain of the excitatory corticothalamic loop is lower in unresponsive wakefulness syndrome (UWS) than in frontal, parietal, and whole-brain conditions; the corticothalamic delay is lower in UWS than in minimally conscious state (MCS) in parietal and whole-brain recordings; the EMG component amplitude is larger in UWS than in MCS across all regions.

**Table D. Statistical comparison of model parameters between the Time Since Onset (TSO) more than 1 year and the TSO less than 1 year groups in both datasets.**

|  |  | $\boldsymbol{G}_{\boldsymbol{ee}}$ | $\boldsymbol{G}_{\boldsymbol{ei}}$ | $\boldsymbol{G}_{\boldsymbol{ese}}$ | $\boldsymbol{G}_{\boldsymbol{esre}}$ | $\boldsymbol{G}_{\boldsymbol{srs}}$ | $\boldsymbol{\alpha}$ | $\boldsymbol{\beta}$ | $\boldsymbol{t}_{\boldsymbol{0}}$ | $\boldsymbol{P}_{\boldsymbol{EMG}}$ |
| --- | --- | --- | --- | --- | --- | --- | --- | --- | --- | --- |
| **Liège** | ***U*-value** | 4059 | 3894 | 4299 | 3714 | 3899 | 4204 | 4124 | 3888 | 4052 |
|  | ***p*-value** | 0.643 | 0.643 | 0.428 | 0.487 | 0.643 | 0.487 | 0.643 | 0.643 | 0.643 |
| **Paris** | ***U*-value** | 10058 | 9704 | 10280 | 8709 | 9187 | 10939 | 11571 | 9217 | 10466 |
|  | ***p*-value** | 0.723 | 0.965 | 0.587 | 0.421 | 0.587 | 0.376 | 0.075 | 0.587 | 0.587 |

**Table E. Correlation between model parameters and CRS-R scores.**

|  |  | $\boldsymbol{G}_{\boldsymbol{ee}}$ | $\boldsymbol{G}_{\boldsymbol{ei}}$ | $\boldsymbol{G}_{\boldsymbol{ese}}$ | $\boldsymbol{G}_{\boldsymbol{esre}}$ | $\boldsymbol{G}_{\boldsymbol{srs}}$ | $\boldsymbol{\alpha}$ | $\boldsymbol{\beta}$ | $\boldsymbol{t}_{\boldsymbol{0}}$ | $\boldsymbol{P}_{\boldsymbol{EMG}}$ |
| --- | --- | --- | --- | --- | --- | --- | --- | --- | --- | --- |
| **Liège** | ***ρ*** | -0.044 | 0.120 | 0.237 | -0.254 | -0.094 | -0.068 | -0.042 | -0.081 | -0.063 |
|  | ***p*-value** | 0.658 | 0.617 | 0.051 | 0.051 | 0.651 | 0.651 | 0.658 | 0.651 | 0.651 |
| **Paris** | ***ρ*** | -0.057 | 0.058 | 0.009 | 0.024 | -0.053 | 0.008 | 0.043 | 0.027 | -0.016 |
|  | ***p*-value** | 0.291 | 0.285 | 0.870 | 0.659 | 0.325 | 0.883 | 0.423 | 0.613 | 0.767 |

**Table F. Comparison of the complexity measure for frontal and parietal channels across groups (Kruskal-wallis test).** We computed the difference in complexity measures between frontal and parietal channels and performed a Kruskal-Wallis test across the three subject groups. No significant differences were found for any of the three measures, indicating that signal complexity between frontal and parietal recordings did not differ across groups. HC: healthy control; MCS: minimally conscious state; UWS: unresponsive wakefulness syndrome. LZC: Lempel-ziv complexity; SE: spectral entropy; PE: permutation entropy.

|  |  | **LZC** | | **SE** | | **PE** | |
| --- | --- | --- | --- | --- | --- | --- | --- |
|  |  | $\boldsymbol{\chi}^{\mathbf{2}}$ | **p-value** | $\boldsymbol{\chi}^{\mathbf{2}}$ | **p-value** | $\boldsymbol{\chi}^{\mathbf{2}}$ | **p-value** |
| **Liège** | **Frontal** | 2.254 | 0.324 | 17.483 | <0.001 | 58.611 | <0.001 |
|  | **Parietal** | 2.203 | 0.332 | 19.212 | <0.001 | 61.268 | <0.001 |
|  | **Difference** | 0.956 | 0.620 | 4.587 | 0.101 | 4.58 | 0.101 |
| **Paris** | **Frontal** | 31.5 | <0.001 | 40.832 | <0.001 | 102.496 | <0.001 |
|  | **Parietal** | 23.9 | <0.001 | 31.882 | <0.001 | 102.197 | <0.001 |
|  | **Difference** | 0.902 | 0.637 | 0.598 | 0.742 | 0.729 | 0.695 |

**Table G. Pair comparison for complexity measures in frontal and parietal channels for real recordings and simulation of two datasets (Mann-Whitney U-test)**. HC: healthy control; MCS: minimally conscious state; UWS: unresponsive wakefulness syndrome. LZC: Lempel-ziv complexity; SE: spectral entropy; PE: permutation entropy.

|  |  |  |  | **LZC** | | **SE** | | **PE** | |
| --- | --- | --- | --- | --- | --- | --- | --- | --- | --- |
|  |  |  |  | **U-value** | **p-value** | **U-value** | **p-value** | **U-value** | **p-value** |
| **Liège** | **Recordings** | **Frontal** | **HC-MCS** | 2526 | 0.202 | 2989 | <0.001 | 3585 | <0.001 |
|  |  |  | **HC-UWS** | 1264 | 0.564 | 1654 | <0.001 | 1836 | <0.001 |
|  |  |  | **MCS-UWS** | 4975 | 0.273 | 5004 | 0.356 | 1833 | 0.193 |
|  |  | **Parietal** | **HC-MCS** | 2554 | 0.152 | 2944 | <0.001 | 3517 | <0.001 |
|  |  |  | **HC-UWS** | 1362 | 0.860 | 1707 | <0.001 | 1862 | <0.001 |
|  |  |  | **MCS-UWS** | 5064 | 0.344 | 5327 | 0.323 | 1543 | 0.003 |
|  | **Simulation** | **Frontal** | **HC-MCS** | 3042 | <0.001 | 3170 | <0.001 | 3698 | <0.001 |
|  |  |  | **HC-UWS** | 1518 | 0.016 | 1573 | 0.002 | 1857 | <0.001 |
|  |  |  | **MCS-UWS** | 4821 | 0.045 | 4804 | 0.035 | 4870 | 0.086 |
|  |  | **Parietal** | **HC-MCS** | 3198 | <0.001 | 3161 | <0.001 | 3656 | <0.001 |
|  |  |  | **HC-UWS** | 1546 | 0.006 | 1509 | 0.022 | 1842 | <0.001 |
|  |  |  | **MCS-UWS** | 4751 | 0.015 | 4699 | 0.006 | 4868 | 0.084 |
| **Paris** | **Recordings** | **Frontal** | **HC-MCS**  **HC-UWS** | 6151 | <0.001 | 6255 | <0.001 | 7110 | <0.001 |
|  |  |  |  | 5474 | <0.001 | 5811 | <0.001 | 6854 | <0.001 |
|  |  |  | **MCS-UWS** | 32948 | 0.765 | 28937 | 0.198 | 25775 | <0.001 |
|  |  | **Parietal** | **HC-MCS** | 5875 | <0.001 | 5897 | <0.001 | 7110 | <0.001 |
|  |  |  | **HC-UWS**  **MCS-UWS** | 5259 | <0.001 | 5685 | <0.001 | 6844 | <0.001 |
|  |  |  |  | 32213 | 0.643 | 29138 | 0.281 | 25772 | <0.001 |
|  | **Simulation** | **Frontal** | **HC-MCS**  **HC-UWS** | 5948 | <0.001 | 6110 | <0.001 | 7268 | <0.001 |
|  |  |  |  | 5629 | <0.001 | 5834 | <0.001 | 6840 | <0.001 |
|  |  |  | **MCS-UWS** | 32862 | 0.834 | 29495 | 0.480 | 28413 | 0.067 |
|  |  | **Parietal** | **HC-MCS** | 5738 | <0.001 | 6021 | <0.001 | 7268 | <0.001 |
|  |  |  | **HC-UWS**  **MCS-UWS** | 5454 | <0.001 | 5819 | <0.001 | 6856 | <0.001 |
|  |  |  |  | 33075 | 0.667 | 29162 | 0.293 | 28015 | 0.025 |

**Fig C. Group comparisons of complexity measures in real recordings and simulations from the Liège dataset.** For sample entropy (SE) and permutation entropy (PE), signal complexity was higher in the healthy control group compared to the patient groups (MCS and UWS) in both real recordings and simulations, consistently across frontal and parietal channels. No significant differences were found between MCS and UWS groups for these two measures. For Lempel-Ziv complexity (LZC), no group differences were observed in the real recordings. However, in the simulations, LZC was higher in healthy controls compared to patient groups in both frontal and parietal channels. Additionally, for the parietal channels, LZC was significantly higher in the UWS group compared to the MCS group. HC: healthy control; MCS: minimally conscious state; UWS: unresponsive wakefulness syndrome.

**Fig D. Group comparison for complexity measures in real recording and simulation of Paris dataset.** Across all three complexity measures and channel conditions, signal complexity was consistently higher in the healthy control group compared to the patient groups, in both real recordings and simulations. Additionally, PE was higher in the MCS group than in the UWS group for frontal and parietal channels in the real recordings, as well as for parietal channels in the simulations. HC: healthy control; MCS: minimally conscious state; UWS: unresponsive wakefulness syndrome. LZC: Lempel-ziv complexity; SE: spectral entropy; PE: permutation entropy.

**Table H. The correlation analyses (Spearman’s coefficient) for Fz and Pz channels of three complexity measures (LZC, SE and PE) in Liège and Paris datasets.** HC: healthy control; MCS: minimally conscious state; UWS: unresponsive wakefulness syndrome. LZC: Lempel-ziv complexity; SE: spectral entropy; PE: permutation entropy.

|  |  |  | **LZC** | | **SE** | | **PE** | |
| --- | --- | --- | --- | --- | --- | --- | --- | --- |
|  |  |  | ***ρ*** | ***p*-value** | ***ρ*** | ***p*-value** | ***ρ*** | ***p*-value** |
| **Liège** | **Frontal** | **HC** | 0.669 | <0.001 | 0.594 | <0.001 | 0.903 | <0.001 |
|  |  | **MCS** | 0.625 | <0.001 | 0.700 | <0.001 | 0.820 | <0.001 |
|  |  | **UWS** | 0.261 | 0.063 | 0.492 | 0.004 | 0.485 | 0.004 |
|  | **Parietal** | **HC** | 0.762 | <0.001 | 0.680 | <0.001 | 0.825 | <0.001 |
|  |  | **MCS** | 0.550 | <0.001 | 0.694 | <0.001 | 0.758 | <0.001 |
|  |  | **UWS** | 0.343 | 0.016 | 0.576 | <0.001 | 0.389 | 0.025 |
| **Paris** | **Frontal** | **HC** | 0.644 | <0.001 | 0.764 | <0.001 | 0.805 | <0.001 |
|  |  | **MCS** | 0.484 | <0.001 | 0.602 | <0.001 | 0.682 | <0.001 |
|  |  | **UWS** | 0.313 | <0.001 | 0.466 | <0.001 | 0.598 | <0.001 |
|  | **Parietal** | **HC** | 0.715 | <0.001 | 0.740 | <0.001 | 0.712 | <0.001 |
|  |  | **MCS** | 0.394 | <0.001 | 0.544 | <0.001 | 0.693 | <0.001 |
|  |  | **UWS** | 0.277 | <0.001 | 0.385 | <0.001 | 0.544 | <0.001 |

**Table I. Statistical comparison of complexity measures between the Time Since Onset (TSO) more than 1 year and the TSO less than 1 year groups in both datasets.** In Liège dataset, the SE value is higher in the TSO>1 group than in the TSO<1 group in simulation; and the PE value is higher in the TSO>1 group in both real recording and simulation. In Paris dataset, for all three measures in both real recording and model simulation, the signal complexity of the TSO>1 group is higher than the TSO<1 group. LZC: Lempel-ziv complexity; SE: spectral entropy; PE: permutation entropy.

|  |  | **LZC** | | **SE** | | **PE** | |
| --- | --- | --- | --- | --- | --- | --- | --- |
|  |  | **U-value** | **p-value** | **U-value** | **p-value** | **U-value** | **p-value** |
| **Liège** | **Real recording** | 4222 | 0.132 | 4202 | 0.166 | 4342 | **0.025** |
|  | **Simulation** | 4239 | 0.107 | 4325 | **0.032** | 4319 | **0.035** |
| **Paris** | **Real recording** | 13238 | **<0.001** | 13167 | **<0.001** | 12480 | **<0.001** |
|  | **Simulation** | 11443 | **0.014** | 11512 | **0.011** | 11659 | **0.006** |

**Fig E. Comparison of complexity measure values between the Time Since Onset (TSO) more than 1 year and the TSO less than 1 year groups in both datasets.** Blue lines indicate a significance level of p<0.05.

**Table J. Correlation between three complexity measures and CRS-R scores in both datasets.** Significant correlations were found in PE value of the Paris dataset in both real recording and simulation. LZC: Lempel-ziv complexity; SE: spectral entropy; PE: permutation entropy.

|  |  | **LZC** | | **SE** | | **PE** | |
| --- | --- | --- | --- | --- | --- | --- | --- |
|  |  | ***ρ*** | **p-value** | ***ρ*** | **p-value** | ***ρ*** | **p-value** |
| **Liège** | **Real recording** | -0.034 | 0.721 | 0.070 | 0.459 | 0.016 | 0.867 |
|  | **Simulation** | -0.073 | 0.440 | -0.051 | 0.589 | 0.018 | 0.848 |
| **Paris** | **Real recording** | 0.019 | 0.883 | 0.098 | 0.158 | 0.290 | **<0.001** |
|  | **Simulation** | 0.058 | 0.504 | 0.108 | 0.137 | 0.176 | **0.005** |
