# Supplementary material for: Individualised electrophysiological neural field models for the assessment of thalamocortical function in disorders of consciousness: A multicentre study
Source: PLoS Comput Biol. 2026 Oct 28;22(10):e1014905. doi: 10.1371/journal.pcbi.1014905 (PMC13730432; doi:10.1371/journal.pcbi.1014905)
Supplement: Peer review history

# Peer review: Decision Letter 0

**DOI:** 10.1371/journal.pcbi.1014905.r001

**Authors:** Hugues Berry, Christian Keitel

20 May 2026

PCOMPBIOL-D-25-02511

Individualised electrophysiological neural field models for the assessment of thalamocortical function in Disorders of Consciousness: a multicentre study.

PLOS Computational Biology

Dear Dr. cóngyǔ,

Thank you for submitting your manuscript to PLOS Computational Biology. After careful consideration, we feel that it has merit but does not fully meet PLOS Computational Biology's publication criteria as it currently stands. Therefore, we invite you to submit a revised version of the manuscript that addresses the points raised during the review process.

Please submit your revised manuscript by Jul 20 2026 11:59PM. If you will need more time than this to complete your revisions, please reply to this message or contact the journal office at ploscompbiol@plos.org. When you're ready to submit your revision, log on to https://www.editorialmanager.com/pcompbiol/ and select the 'Submissions Needing Revision' folder to locate your manuscript file.

Please include the following items when submitting your revised manuscript:

* A letter that responds to each point raised by the editor and reviewer(s). You should upload this letter as a separate file labeled 'Response to Reviewers'. This file does not need to include responses to formatting updates and technical items listed in the 'Journal Requirements' section below.

* A marked-up copy of your manuscript that highlights changes made to the original version. You should upload this as a separate file labeled 'Revised Manuscript with Track Changes'.

* An unmarked version of your revised paper without tracked changes. You should upload this as a separate file labeled 'Manuscript'.

If you would like to make changes to your financial disclosure, competing interests statement, or data availability statement, please make these updates within the submission form at the time of resubmission. Guidelines for resubmitting your figure files are available below the reviewer comments at the end of this letter.

As the corresponding author, your ORCID iD is verified in the submission system and will appear in the published article. PLOS supports the use of ORCID, and we encourage all coauthors to register for an ORCID iD and use it as well. Please encourage your coauthors to verify their ORCID iD within the submission system before final acceptance, as unverified ORCID iDs will not appear in the published article. Only the individual author can complete the verification step; PLOS staff cannot verify ORCID iDs on behalf of authors.

We look forward to receiving your revised manuscript.

Kind regards,

Christian Keitel

Academic Editor

PLOS Computational Biology

Hugues Berry

Section Editor

PLOS Computational Biology

**Additional Editor Comments:**

Please accept my apologies for the long wait time. It turned out extremely difficult and long-winding to find reviewers for your submission but your submission has now been reviewed by two experts and they both see merit in the work. I ask you to consider all their comments carefully when crafting a revision of the work. I'd like to highlight Rev 1's request to better highlight and detail the "key spectral features" reproduced by the model, as well as Rev 2's comment on the spatial specificity of the modelling, both of which seem essential to address.

**Journal Requirements:**

If the reviewer comments include a recommendation to cite specific previously published works, please review and evaluate these publications to determine whether they are relevant and should be cited. There is no requirement to cite these works unless the editor has indicated otherwise.

1) Please ensure that the CRediT author contributions listed for every co-author are completed accurately and in full.

At this stage, the following Authors/Authors require contributions: lín cóngyǔ, Naji L.N. Alnagger, Jitka Annen, Laouen Belloli, Olivia Gosseries, Steven Laureys, Iván Mindlin, Lionel Naccache, Pablo Núñez, Benjamin Rohaut, Jacobo D. Sitt, Prejaas Tewarie, Aurore Thibaut, and Glenn J.M. Van der Lande. Please ensure that the full contributions of each author are acknowledged in the "Add/Edit/Remove Authors" section of our submission form.

The list of CRediT author contributions may be found here: https://journals.plos.org/ploscompbiol/s/authorship#loc-author-contributions

2) We do not publish any copyright or trademark symbols that usually accompany proprietary names, eg ©,  ®, or TM  (e.g. next to drug or reagent names). Therefore please remove all instances of trademark/copyright symbols throughout the text, including:

- ® on page: 19.

3) Please upload all main figures as separate Figure files in .tif or .eps format. For more information about how to convert and format your figure files please see our guidelines:

https://journals.plos.org/ploscompbiol/s/figures

4) We have noticed that you have uploaded Supporting Information files, but you have not included a list of legends. Please add a full list of legends for your Supporting Information files after the references list.

5)  Please ensure that the funders and grant numbers match between the Financial Disclosure field and the Funding Information tab in your submission form. Note that the funders must be provided in the same order in both places as well.

**Reviewers' comments:**

Reviewer's Responses to Questions

**Comments to the Authors:**

**Please note here if the review is uploaded as an attachment.**

Reviewer #1: Congyu and colleagues propose an interesting analysis of how neural field models that have been fit to reproduce spectral features of non-invasive brain recording can then produce other partially unrelated features of recordings (complexity metrics) and even allow some mechanistic hypothesis testing through the study of the fit parameters in different populations. The paper is well written and the presented analyses are rigorous. The application is interesting. Nonetheless, I have some comments for the author to address before proceeding further.

- The current version of Figure 1 is very small... Maybe putting panels A and B one below the other could help readability? I also feel that panel B could be more explicit on model details thus allowing the reader to have a better idea of what kind of model we are talking about without the need to go and look in the Methods.

- In the Discussion the authors say that "the corticothalamic model [...] captured key spectral features" but from Figure 2 and the corresponding text in the results I do not see adequate numerical justification to this. I would like to see a more detailed treatment of the salient reproduced features mentioned in discussion. This holds also for other observations throughout the discussion, like comments on high fit parameter standard deviations etc.

- Following on this point, it is not obvious to me how to interpret the spectra in Figure 2. More detailed explanation of the difference between "spectral model" and "timeseries" and the important different between "timeseries" and the other shown spectra should be added in the main text, in particular in the Results section. Again, more granular numerical results than the relatively "abstract" Chi square values would be very helpful.

- Throughout the text, there are a lot of abbreviations that are either defined multiple times, or never used again. I believe that a more coherent use of abbreviations (and maybe the definition of less abbreviations altogether) would benefit readability. Following on this, the results section is at times very dense and a little difficult to follow.

- The introduction and discussion sections are well structured but I am missing a more clear positioning of the presented research. The clinical considerations opening the manuscript suggest me that the final goal of this research may be of translational / clinical interest but I do not see an at least tentative way forward. Of course there is no strong need of being a clinically oriented work to proceed to publication, but I would like to read a clearer declaration of intent: translational / clinical vs. neuroscientific / fundamental.

- I would appreciate to see a well defined paragraph on the novelty of the study. The discussion is quite well-written and documented but at times it risks to feel like we already knew / expected most of the nice results in the manuscript.

- Please pay attention at some typos here and there throughout the manuscript. I have also seen a broken internal link that should be fixed.

Reviewer #2: The objective of the paper is to distinguish neuronal differences in disorders of consciousness using a neural field model. The authors consider two EEG datasets acquired under different conditions. The model parameters are optimized to simulate spectral features across different states of consciousness. Results suggest reduced corticothalamic integrity in patients.

I have two major concerns regarding the modeling strategy and the use of dataset.

- Modeling:

If I understood connrectly, the neural field model is fitted to EEG signals averaged across 183 electrodes (or averaged within frontal/parietal subsets), effectively representing large-scale brain activity with a highly reduced single-node description. While this makes the fitting procedure more tractable, it also removes much of the spatial and spatiotemporal structure that is likely relevant in disorders of consciousness. In particular, the approach cannot capture regional differences, network interactions, or propagation effects that may differ between MCS and UWS patients.

The rationale for reducing the dimensionality of the problem is understandable, but the limitations of this simplification should be discussed more explicitly. At present, the physiological interpretation of the inferred corticothalamic parameters are overstated given the very coarse spatial representation of the data. I think it is important to comment on whether future extensions of the model could incorporate structural connectivity or multi-node thalamo-cortical architectures while still allowing parameter estimation from EEG data. The first step can be simulating EEG signals from Fz and Pz with a model where there is only one thalamus and the brain regions representing Fz and Pz are communicating directly (corto-cortico connection) and indirectly (cortico-thalamo-cortico conneciton).

- Dataset:

Two datasets acquired under substantially different experimental conditions. The Liège dataset consists of resting-state EEG recordings, whereas the Paris dataset was obtained during an auditory local-global paradigm and later treated as a “pseudo-resting-state” condition. Since the local-global paradigm is itself designed to probe conscious processing, it is difficult to disentangle effects related to the experimental condition from effects related to the level of consciousness.

This issue is important because several of the key findings distinguishing MCS from UWS appear primarily in the Paris dataset, while they are absent or inconsistent in the Liège resting-state dataset. As a results, some of the reported differences in fitted parameters may may partly reflect paradigm-dependent neural dynamics rather than stable biomarkers of consciousness level. Although the authors acknowledge this limitation briefly in the Discussion, I think that it deserves substantially more emphasis earlier in the manuscript, particularly when interpreting between-dataset differences and discussing the generalizability of the results.

- Minor remark:

Line 223: I guess that it is Figure1A that shows a a schematic description of the main steps of the study, while Figure 1B shows the model.

**********

**Have the authors made all data and (if applicable) computational code underlying the findings in their manuscript fully available?**

The PLOS Data policy requires authors to make all data and code underlying the findings described in their manuscript fully available without restriction, with rare exception (please refer to the Data Availability Statement in the manuscript PDF file). The data and code should be provided as part of the manuscript or its supporting information, or deposited to a public repository. For example, in addition to summary statistics, the data points behind means, medians and variance measures should be available. If there are restrictions on publicly sharing data or code —e.g. participant privacy or use of data from a third party—those must be specified.

Reviewer #1: Yes

Reviewer #2: Yes

**********

PLOS authors have the option to publish the peer review history of their article (what does this mean?). If published, this will include your full peer review and any attached files.

If you choose “no”, your identity will remain anonymous but your review may still be made public.

**Do you want your identity to be public for this peer review?**  For information about this choice, including consent withdrawal, please see our Privacy Policy.

Reviewer #1: No

Reviewer #2: No

**Figure resubmission:**

While revising your submission, we strongly recommend that you use PLOS’s NAAS tool (https://ngplosjournals.pagemajik.ai/artanalysis) to test your figure files. NAAS can convert your figure files to the TIFF file type and meet basic requirements (such as print size, resolution), or provide you with a report on issues that do not meet our requirements and that NAAS cannot fix.

After uploading your figures to PLOS’s NAAS tool - https://ngplosjournals.pagemajik.ai/artanalysis, NAAS will process the files provided and display the results in the "Uploaded Files" section of the page as the processing is complete. If the uploaded figures meet our requirements (or NAAS is able to fix the files to meet our requirements), the figure will be marked as "fixed" above. If NAAS is unable to fix the files, a red "failed" label will appear above. When NAAS has confirmed that the figure files meet our requirements, please download the file via the download option, and include these NAAS processed figure files when submitting your revised manuscript.

**Reproducibility:**

To enhance the reproducibility of your results, we recommend that authors of applicable studies deposit laboratory protocols in protocols.io, where a protocol can be assigned its own identifier (DOI) such that it can be cited independently in the future. Additionally, PLOS ONE offers an option to publish peer-reviewed clinical study protocols. Read more information on sharing protocols at https://plos.org/protocols?utm_medium=editorial-email&utm_source=authorletters&utm_campaign=protocols

# Author comment: Author response to Decision Letter 1

**DOI:** 10.1371/journal.pcbi.1014905.r002

10 Aug 2026

Attachment Submitted filename: reply to reviewers.pdf

# Peer review: Decision Letter 1

**DOI:** 10.1371/journal.pcbi.1014905.r003

**Authors:** Hugues Berry, Christian Keitel

15 Oct 2026

Dear Ms. cóngyǔ,

We are pleased to inform you that your manuscript 'Individualised electrophysiological neural field models for the assessment of thalamocortical function in disorders of consciousness: a multicentre study.' has been provisionally accepted for publication in PLOS Computational Biology.

Before your manuscript can be formally accepted you will need to complete some formatting changes, which you will receive in a follow up email. A member of our team will be in touch with a set of requests.

Please note that your manuscript will not be scheduled for publication until you have made the required changes, so a swift response is appreciated.

IMPORTANT: The editorial review process is now complete. PLOS will only permit corrections to spelling, formatting or significant scientific errors from this point onwards. Requests for major changes, or any which affect the scientific understanding of your work, will cause delays to the publication date of your manuscript.

Should you, your institution's press office or the journal office choose to press release your paper, you will automatically be opted out of early publication. We ask that you notify us now if you or your institution is planning to press release the article. All press must be co-ordinated with PLOS.

Thank you again for supporting Open Access publishing; we are looking forward to publishing your work in PLOS Computational Biology.

Best regards,

Christian Keitel

Academic Editor

PLOS Computational Biology

Hugues Berry

Section Editor

PLOS Computational Biology

***********************************************************

Dear authors, the reviewers have found that all their comments have been addressed. In line with the final remark of one of the two reviewers, please make sure to check whether you have made all necessary materials available to allow others to reproduce your results.

Reviewer's Responses to Questions

**Comments to the Authors:**

**Please note here if the review is uploaded as an attachment.**

Reviewer #1: The authors have addressed my comments

Reviewer #2: Authors replied my concerns.

**********

**Have the authors made all data and (if applicable) computational code underlying the findings in their manuscript fully available?**

The PLOS Data policy requires authors to make all data and code underlying the findings described in their manuscript fully available without restriction, with rare exception (please refer to the Data Availability Statement in the manuscript PDF file). The data and code should be provided as part of the manuscript or its supporting information, or deposited to a public repository. For example, in addition to summary statistics, the data points behind means, medians and variance measures should be available. If there are restrictions on publicly sharing data or code —e.g. participant privacy or use of data from a third party—those must be specified.

Reviewer #1: Yes

Reviewer #2: **No:**  They provided the tools but not the full implementation.

**********

PLOS authors have the option to publish the peer review history of their article (what does this mean?). If published, this will include your full peer review and any attached files.

If you choose “no”, your identity will remain anonymous but your review may still be made public.

**Do you want your identity to be public for this peer review?**  For information about this choice, including consent withdrawal, please see our Privacy Policy.

Reviewer #1: No

Reviewer #2: No

# Editor report: Acceptance letter

**DOI:** 10.1371/journal.pcbi.1014905.r004

**Authors:** Hugues Berry, Christian Keitel

PCOMPBIOL-D-25-02511R1

Individualised electrophysiological neural field models for the assessment of thalamocortical function in disorders of consciousness: a multicentre study.

Dear Dr cóngyǔ,

I am pleased to inform you that your manuscript has been formally accepted for publication in PLOS Computational Biology. Your manuscript is now with our production department and you will be notified of the publication date in due course.

The corresponding author will soon be receiving a typeset proof for review, to ensure errors have not been introduced during production. Please review the PDF proof of your manuscript carefully, as this is the last chance to correct any errors. Please note that major changes, or those which affect the scientific understanding of the work, will likely cause delays to the publication date of your manuscript.

Soon after your final files are uploaded, unless you have opted out, the early version of your manuscript will be published online. The date of the early version will be your article's publication date. The final article will be published to the same URL, and all versions of the paper will be accessible to readers.

For Research, Software, and Methods articles, you will receive an invoice from PLOS for your publication fee after your manuscript has reached the completed accept phase. If you receive an email requesting payment before acceptance or for any other service, this may be a phishing scheme. Learn how to identify phishing emails and protect your accounts at https://explore.plos.org/phishing.

Thank you again for supporting PLOS Computational Biology and open-access publishing. We are looking forward to publishing your work!

With kind regards,

Sharmila Kamatchi

PLOS Computational Biology | Carlyle House, Carlyle Road, Cambridge CB4 3DN | United Kingdom ploscompbiol@plos.org | Phone +44 (0) 1223-442824 | ploscompbiol.org | @PLOSCompBiol
